# A Double Swath Configuration for Improving Throughput and Accuracy of Trait Estimate from UAV Images

**DOI:** 10.34133/2021/9892647

**Published:** 2021-12-06

**Authors:** Wenjuan Li, Alexis Comar, Marie Weiss, Sylvain Jay, Gallian Colombeau, Raul Lopez-Lozano, Simon Madec, Frédéric Baret

**Affiliations:** ^1^HIPHEN, 228 Route de l'Aérodrome, 84000 Avignon, France; ^2^INRAE, Avignon Université, UMR EMMAH, 84000 Avignon, France; ^3^ARVALIS Institut du Végétal, 3 Rue Joseph et Marie Hackin, 75116 Paris, France

## Abstract

Multispectral observations from unmanned aerial vehicles (UAVs) are currently used for precision agriculture and crop phenotyping applications to monitor a series of traits allowing the characterization of the vegetation status. However, the limited autonomy of UAVs makes the completion of flights difficult when sampling large areas. Increasing the throughput of data acquisition while not degrading the ground sample distance (GSD) is, therefore, a critical issue to be solved. We propose here a new image acquisition configuration based on the combination of two focal length (*f*) optics: an optics with *f* = 4.2 mm is added to the standard *f* = 8 mm (SS: single swath) of the multispectral camera (DS: double swath, double of the standard one). Two flights were completed consecutively in 2018 over a maize field using the AIRPHEN multispectral camera at 52 m altitude. The DS flight plan was designed to get 80% overlap with the 4.2 mm optics, while the SS one was designed to get 80% overlap with the 8 mm optics. As a result, the time required to cover the same area is halved for the DS as compared to the SS. The georeferencing accuracy was improved for the DS configuration, particularly for the *Z* dimension due to the larger view angles available with the small focal length optics. Application to plant height estimates demonstrates that the DS configuration provides similar results as the SS one. However, for both the DS and SS configurations, degrading the quality level used to generate the 3D point cloud significantly decreases the plant height estimates.

## 1. Introduction

Since recent years, unmanned aerial vehicles (UAVs) have become very popular to estimate several crop traits for application to precision agriculture [1, 2] and high-throughput plant phenotyping [3]. They can be operated in a near real-time and dynamic manner and are relatively low-cost [4]. Significant progress has been achieved to extract traits such as crop height, the cover fraction, green area index (GAI), or chlorophyll and nitrogen contents from different optical sensors [5–9]. Among these optical sensors, multispectral cameras working in the visible (400-700 nm) and near-infrared (700-1000 nm) spectral domain are well suited for vegetation monitoring [10, 11]. The images can be either used to calculate vegetation indices directly [5] or to estimate canopy structural traits such as GAI [8, 12] and biochemical traits such as chlorophyll content [13]. Despite remarkable research and applications, there are still some bottlenecks that limit the efficiency and accuracy of multispectral cameras onboard UAVs.

The operational use of UAVs is limited by the battery capacity, which constrains flight duration and thus the throughput. A possible way to overcome this issue is to increase the flight altitude but at the expense of degradation of the spatial resolution, which may be critical for estimating some traits [14]. Another way is to decrease the overlap between images although this may degrade the quality of the orthomosaic and 3D description of the surface from the structure from motion algorithm [6]. A compromise must therefore be found between the data acquisition throughput, spatial resolution at the ground level, and the accuracy of the 3D scene reconstruction of the surface [15].

Structure from motion (SfM) algorithms are increasingly used to reconstruct the 3D geometry from overlapping imagery [16]. SfM is widely used to estimate plant height, which is a proxy for biomass estimation [6, 14, 17, 18] and allows quantifying lodging [19]. Several studies demonstrated the potential of SfM methods for plant height estimation [6, 15, 20, 21]. However, some difficulties may be encountered when the diversity of points of view for each place on the ground is limited. This can be improved by combining nadir and oblique view directions [22], either by flying two cameras with different fields of view [6] or inclined differently. Alternatively, at the expense of flight duration and throughput, the camera can also fly twice with one flight for nadir viewing and the other for oblique viewing [23].

This paper presents a novel method that is aimed at improving the image acquisition throughput and the processing for a given targeted spatial resolution while possibly improving the quality of the 3D surface reconstruction. It is based on the combination of at least two cameras with different focal lengths. It provides thus (at least) two different swaths and is called double swath (DS) for this reason. It is compared to the current way to acquire and process images using a single focal length providing therefore a single swath and will be called SS for this reason. A dedicated field experiment was conducted to evaluate the efficiency of this method against a classical SS flight configuration. A multispectral camera was used here to illustrate the principles and the possible impact on the geometric accuracy, the 3D surface description including plant height estimation, the data acquisition throughput, and the processing time.

## 2. Materials and Methods

### 2.1. Study Area

**Figure 1:**
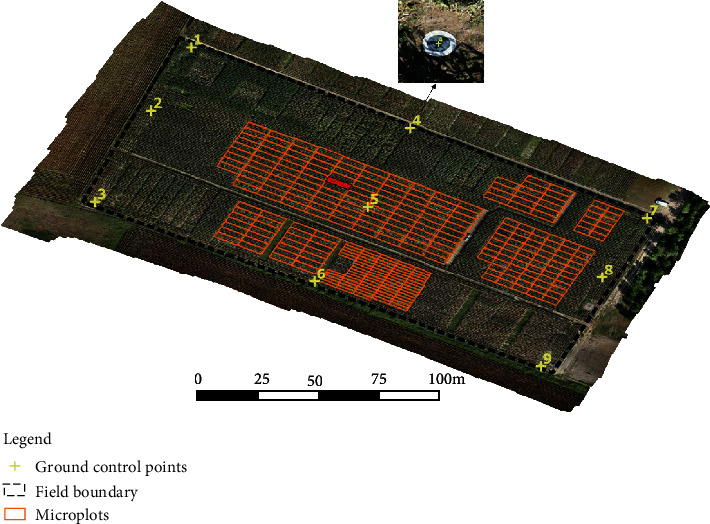
RGB orthoimage of the experimental field. Ground control points (#1~9) are indicated with yellow crosses, and the boundaries of the 299 microplots studied are shown in orange. The black dashed line delimits the studied area. The microplot highlighted in red in the center of the field is used to calculate the view angles of the camera in Section 3.1. One screenshot of GCP #4 was added after zooming in the orthoimage with scale 1 : 23 in QGIS software.

An experimental field of 2.2 ha area located in the southwest of France (Onard, 43°47′ N, 0°48′ W, Figure 1) included 827 microplots with different maize genotypes sown in May 2018 and harvested in October 2018. We focused on part of the experimental field, including 227 microplots of 10.5 m × 3.15 m size and 72 of 8 m × 1.6 msize. The average temperature of this region varies from 4.9°C in the winter and 27.2°C in August, with average rainfall from 31 mm in August to 90 mm in November (https://www.weather-atlas.com/en/france/onard-climate#temperature).

### 2.2. Multispectral Camera

The AIRPHEN multispectral camera (https://www.hiphen-plant.com/solutions/airphen/) was fixed on a hexacopter equipped with a two-axis gimbal designed to align the camera vertically downward. The multispectral camera is made of six elementary CMOS cameras with a full width at half maximum close to 10 nm and 1280 × 960 pixels of 3.04 *μ*m size. Five cameras (blue: 450 nm, green: 530 nm, red: 675 nm, red-edge: 730 nm, and near-infrared: 850 nm) were equipped with an 8 mm focal length lens, with a relatively narrow field of view (FOV) of 33° × 25°. The sixth camera (far green: 570 nm) had a 4.2 mm focal length lens with a larger FOV of 60° × 46°. The cameras were oriented such as the larger dimension of the image was across-track, defining the swath of the system. The system is called double swath because of the coexistence of two different swaths as well as because the 4.2 mm focal length camera doubles the swath of the 8 mm ones. The six cameras were synchronized and automatically triggered at a 1.0 Hz frequency. The integration time of each of the six cameras was adjusted automatically to minimize saturation and maximize the dynamics. The images were recorded in a 16-bit tiff format along with acquisition time, camera global positioning system (GPS) coordinates, and integration time.

### 2.3. UAV Flight Plans for Single and Double Swath Configurations

The UAV was flown on September the 18^th^, close to solar noon under clear sky conditions with a solar zenith angle of 42°. Maize plants were fully developed, and senescence was underway. The UAV altitude was set to 52 m above the soil to get a 2.5 cm ground sample distance (GSD) with the 8 mm focal length of the multispectral camera. It was considered sufficient to derive accurate estimates of structural or biochemical traits. The flights were completed within less than one hour.

Two different flight plans were designed to simulate the single swath (SS) and double swath (DS) configurations (Table 1). The SS flight corresponds to the standard configuration where the 8 mm focal length camera is used to provide the required 2.5 cm GSD with 80% overlap both across-track and along-track allowing the SfM algorithm to work efficiently. The DS flight was designed to achieve 80% across-track and along-track overlap of images for the 4.2 mm focal length camera which was the main camera used for the SfM algorithm, with a GSD degraded to 4.8 cm. This resulted in a 62% overlap of images for the 8 mm cameras in both across-track and along-track. Note that the number of images was divided by two for the DS configuration while the flight time was also almost halved (Table 1).

Nine circular panels of 60 cm diameter, placed in the four corners of the field as well as in the center, were used as ground control points (GCPs, see Figure 1) for georeferencing the images and the cameras during the flights as proposed by [24]. These GCPs also allowed us to compute metrics to evaluate the georeferencing accuracy (see Section 2.5.2). The geographic coordinates of GCPs were measured with a real-time kinetics (RTK) GPS device (Trimble Geo 7x) with an accuracy of 1 cm.

### 2.4. Image Processing to Get Plant Height and the Orthoimage

**Figure 2:**
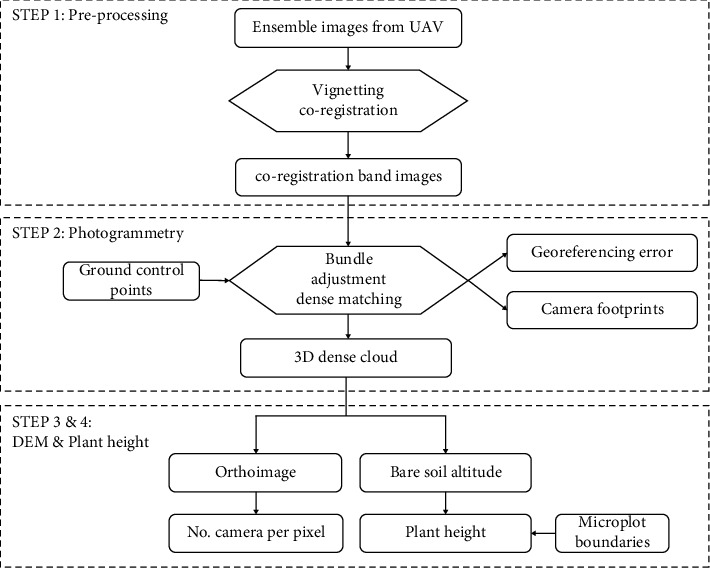
Flowchart of image processing.

The processing chain of the multispectral camera includes four steps (Figure 2). In the first step, some unusable images (e.g., images taken during take-off and landing and blurred images) were firstly discarded. The vignetting effect of cameras was then corrected as described in [8], and the images from the several cameras of the multispectral camera shot with the same trigger were coregistered based on image similarity [25] with accuracy finer than one pixel.

In the second step, *Agisoft Photoscan Professional* edition (Version 1.4.3, Agisoft LLC., Russia) was used to derive the camera position and orientation for each trigger. *Photoscan* is one of the most widely used SfM software [26, 27]. For the SS configuration, blue, green, and red bands acquired with *f* = 8 mm were imported in *Photoscan* as a multispectral project. For the DS configuration, the far green band with *f* = 4.2 mm was imported in addition to the three previous ones to create another multispectral project. These bands were selected to generate an RGB 3D dense cloud to derive plant height. The green band was set as the master band for the SS and DS multispectral projects. For each flight, *Photoscan* generated a set of tie points followed by a bundle adjustment [28, 29] where the GCPs automatically detected were used. After the alignment step, a 3D dense cloud was generated from dense-matching photogrammetry using a moderate depth filtering option and the full image resolution as implemented in *Photoscan*. Three dense clouds were generated with medium, high, and ultrahigh quality levels, respectively. They were used to assess its impact on the quality of the 3D dense cloud and plant height estimation. For ultrahigh resolution, *Photoscan* uses the native image GSD, while for high and medium quality levels, *Photoscan* downscales images to 2 and 4 times the native GSD, respectively. The resulting 3D dense clouds were exported as 8-bit RGB images. At the end of this step, camera footprints were also exported together with the camera position, focal length, and flight altitude.

In the third step, a DEM (Digital Elevation Model) was built from the 3D dense point cloud and then used to create an orthoimage. The number of images on each point of a 0.5 m × 0.5 m grid was finally counted based on the previously exported camera footprints on the orthoimage of each flight.

In the last step, the plant height of each microplot was finally estimated using the method described by [6]. First, the 3D dense cloud corresponding to each microplot was extracted and divided into several consecutive elementary cells of 0.5 m by 0.6 m. This cell size was demonstrated to be large enough to get a good description of the altitude profile including enough ground-level points [6]. In each cell, the *k*-means clustering method [30] with two classes was applied to separate the ground from the vegetation based on both the altitude values and the RGB values as proposed by [31]. The maximum peak in the altitude profile of these ground-level points was considered the soil altitude. The vegetation height was then calculated by subtracting the soil altitude from the original 3D dense cloud for each elementary cell. The final plant height of a microplot was defined as the 99.5% quantile of cumulated height distribution of the vegetation points over all the 0.5 m × 0.6 m cells contained. Three plant heights were calculated from medium, high, and ultrahigh quality of the 3D dense cloud points, respectively.

### 2.5. Efficiency and Quality Assessment

#### 2.5.1. Acquisition and Processing Efficiency

The flight duration, the number of images, and the time required for the photogrammetric processing were the criteria used to evaluate the efficiency of DS and SS configurations. Additional metrics such as the average number of overlapping images on the same ground sampling area and the view angle distribution were also analyzed. Note that the photogrammetric processing was performed on a computer with Intel® Xeon® CPU E5-2630 v3 @ 2.40 GHz and 64 G random-access memory.

#### 2.5.2. Georeferencing Errors

The georeferencing error assesses the absolute true error on geolocation of independent checkpoints, which provides a more robust method to compare DS and SS flights [32]. In this study, georeferencing errors were computed during the bundle adjustment phase (Section 2.4). The georeferencing error was calculated as the residual for each GCP point *i* and each dimension following the expressions:

(1) $$\begin{array}{c} \delta_{X,i}=X_{i}-\hat{X_{i}},\\ \delta_{Y,i}=Y_{i}-\hat{Y_{i}},\\ \delta_{Z,i}=Z_{i}-\hat{Z_{i}}, \end{array}$$

where *X*, *Y*, and *Z* are, respectively, the longitude, latitude, and altitude measured with an RTK GPS instrument and $\hat{X_{i}}$, $\hat{Y_{i}}$, and $\hat{Z_{i}}$ are, respectively, the longitude, latitude, and altitude estimated from *Photoscan*. For the *i*^th^ GCP, the georeferencing error, *ε*_*i*_, was calculated over all the three dimensions using

(2) $$\begin{array}{c} \varepsilon_{i}=\sqrt{\frac{\delta_{X,i}^{2}+\delta_{Y,i}^{2}+\delta_{Z,i}^{2}}{3}}. \end{array}$$

For each dimension, the mean georeferencing error over all GCPs was calculated as

(3) $$\begin{array}{c} \varepsilon^{X}=\sqrt{\frac{\sum {\left(\delta_{X,i}\right)}^{2}}{N}},\\ \varepsilon^{Y}=\sqrt{\frac{\sum {\left(\delta_{Y,i}\right)}^{2}}{N}},\\ \varepsilon^{Z}=\sqrt{\frac{\sum {\left(\delta_{Z,i}\right)}^{2}}{N}}, \end{array}$$

where *N* is the number of GCP points used.

Finally, the overall georeferencing error over the three dimensions and GCPs was calculated as

(4) $$\begin{array}{c} \varepsilon =\sqrt{\frac{\sum_{i}{\varepsilon_{i}}^{2}}{N}}. \end{array}$$

As there were only nine GCPs, the georeferencing error was calculated using a leave-one-out cross-validation, i.e., using *N* − 1 GCPs used for the processing and the remaining GCP used as an independent checkpoint to evaluate the error. This process was repeated over the nine GCPs available.

#### 2.5.3. 3D Dense Cloud and Plant Height Assessment

To assess the differences between the generated 3D dense clouds, the Multiscale Model to Model Cloud Comparison (M3C2) algorithm [33] available within the CloudCompare software (version v2.11.3) was used. It offers a robust cloud change detection procedure that can be used directly on point clouds [34–37]. It computes cloud-to-cloud distance using a local normal direction for each point and fits a cylinder of a specified radius in the direction of the normal vector, instead of considering only the vertical direction. The distance is calculated as the average distance between the two dense clouds in the cylinder, making this algorithm less sensitive to surface noise.

For each quality level, the distances between dense clouds from DS and SS configurations were firstly computed using the M3C2 algorithm, by considering the SS flight as the reference. Spatial distribution and histogram of distances were computed, using median and standard deviation as metrics. Then, for each configuration, the medium and high quality 3D dense clouds were compared with the ultrahigh quality dense cloud, considered here as the reference.

Plant height values were derived from the DS and SS configurations with the multispectral camera computed for the 299 maize microplots highlighted in Figure 1. The coefficient of determination (*R*^2^), root-mean-square error (RMSE), and relative RMSE (RRMSE) were used as metrics to quantify the performances. The impact of the 3D dense cloud quality on plant height estimation was also investigated.

## 3. Results

### 3.1. Acquisition and Processing Efficiency

The flight duration and the number of images taken were halved under DS configuration (Table 2), showing therefore a very high gain for the acquisition efficiency.

The reduction of images to be processed for the DS configuration also explained the significant gain of processing time for all the steps and particularly for the generation of the 3D dense cloud which is generally the most demanding one (Table 2).

**Figure 3:**
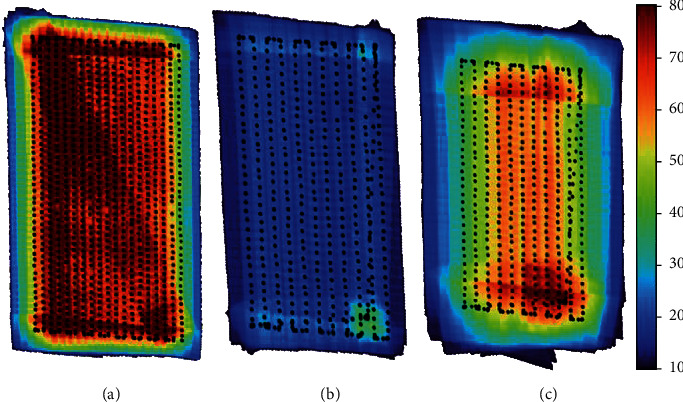
Camera positions (black dots) and number of images per pixel of orthoimage for (a) SS *f* = 8 mm (green band), (b) DS *f* = 8 mm (green band), and (c) DS *f* = 4.2 mm (far green band). The color bar on the right represents the number of images of each pixel of orthoimage that was averaged over 0.5 m × 0.5 m cells.

**Figure 4:**
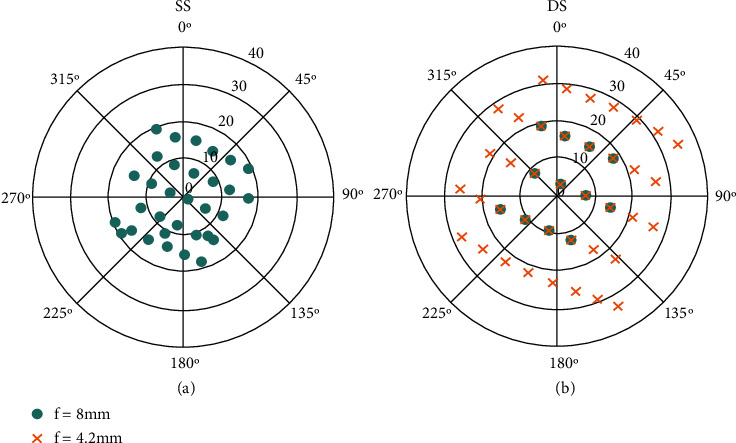
Polar distribution of view angles for one central microplot from SS (a) and DS (b) flights on *f* = 8 mm (green circles) and *f* = 4.2 mm (orange crosses). The central microplot is highlighted in red in Figure 1.

The lower number of images available and the degraded resolution for the 4.2 mm focal length explain the decrease of the number of tie points observed for DS (Table 2). The number of images per pixel was quite different for *f* = 8 mm lens between the SS and DS (Figures 3(a) and 3(b)) because of the reduced overlap between images in the DS configuration (Table 1). However, the camera with *f* = 4.2 mm used for the DS configuration (Figure 3(c)) shows as expected a higher number of images per pixel because of the 80% overlap between images designed for this camera. Finally, the density of points in the 3D dense cloud appeared very comparable between both configurations. For the medium and high quality levels, DS achieved even slightly higher point density as compared to SS. Conversely, for the ultrahigh level, SS provided a slightly higher point density. The larger range of angular distribution observed for the DS configuration due to the 4.2 mm focal length explains the very good 3D dense cloud construction despite the reduced number of images available as compared to SS. The combination of the nadir (zenith angles within 20°) and more oblique views (zenith angles ranging from 0° to 36°) allows increasing the number of images acquired over the same pixel thanks to the 4.2 mm focal length (view zenith angles ranging from 0° to 36°), leading to very similar results in terms of dense point clouds (Figure 4).

### 3.2. Georeferencing Error

The DS configuration shows a smaller overall georeferencing error as compared to the SS one (Table 3). This was evaluated over the orthomosaic constructed at 2.4 cm GSD and ultrahigh quality level. Most of the gain of the DS configuration was coming from the *Z* dimension (Table 3). This agrees with results from [6, 22] who found that larger view angles improve the accuracy of the *Z* component.

A well-accepted absolute value of the georeferencing error is two times the GSD in *X* and *Y* dimensions and three times the GSD in the *Z* dimension [38]. All GCPs from the DS configuration satisfied this requirement, whereas only two GCPs (#1 and #2) had an acceptable error for the SS configuration. Three points (#3, #6, and #9) had large georeferencing errors for the SS configuration, particularly for the *Z* dimension. These three points were located close to the border and were observed therefore from a smaller number of directions and less oblique ones (Table S1).

The georeferencing error was not homogeneous and depended on the distribution of the GCPs used for the georeferencing and the checkpoints used to compute the associated error as we observed (Table 3) in agreement with [36, 38, 39]. The number of GCPs and checkpoints used in the other studies was variable: 21 GCPs and 6 checkpoints in [36], 6 GCPs and 6 checkpoints set in [38], and 19 GCPs and 5 checkpoints in [6]. We only used 9 GCPs evenly distributed in the field with a leave-one-out approach where 8 GCPs are iteratively selected for georeferencing and the remaining one was used as a checkpoint. This approach may lead to an overestimation of the georeferencing error since some checkpoints will not be in between GCPs, with generally higher error as observed in the corners (Table 3 for points [1, 3, 7, 9] displayed in Figure 1).

### 3.3. 3D Dense Clouds

**Figure 5:**
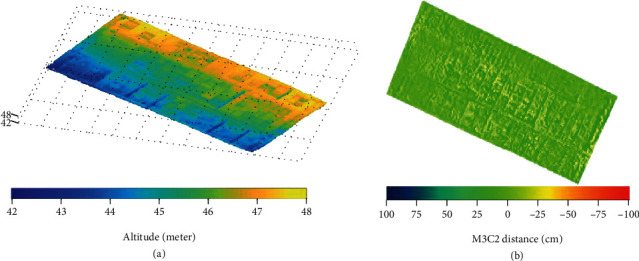
(a) 3D dense clouds computed with ultrahigh quality level for the DS configuration; (b) difference between the ultrahigh quality dense clouds derived from DS and SS flights.

**Figure 6:**
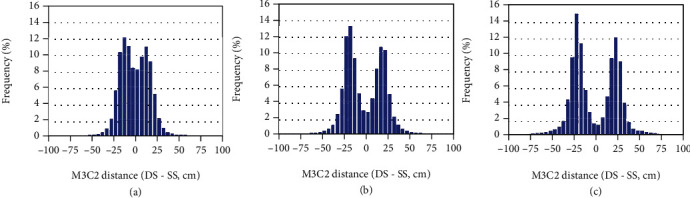
M3C2-calculated distance (cm) between DS and SS configurations: ultrahigh (a), high (b), and medium (c) levels.

All the 3D point clouds show altitudes between 42 m and 48 m, with similar spatial patterns between SS and DS for all the quality levels considered, including higher values in the northeast region (Figure 5(a)). A more detailed inspection shows that 80% of the distance between the 3D dense cloud points of DS and SS configurations is lying within ±20 cm for the ultrahigh level (Figure 6, ultrahigh). The larger differences were observed on the borders where the number of images used to generate the 3D point cloud is lower for the SS configuration. (Figure 5(b)). Moreover, some differences larger than 10 cm were observed at the borders of the microplots (Figure 6). Slight differences in georeferencing of the points between the two configurations may explain this problem in such places with a high gradient in altitude. This also explains the bimodal pattern of the differences observed for all the quality levels (Figure 6).

The quality level used to generate the 3D point clouds impacts the consistency between the SS and DS configurations. Higher quality levels show better agreement (Figure 6). Almost no bias was observed for the ultrahigh quality levels, while it increased when the quality level degraded (Table 4). Broader distribution of the differences was also observed when the quality level degraded (Table 4). The discrepancies between quality levels for each configuration (Table 4) explain the degradation of the consistency between SS and DS 3D point clouds when the quality level decreased (Table 4).

### 3.4. Plant Height

**Figure 7:**
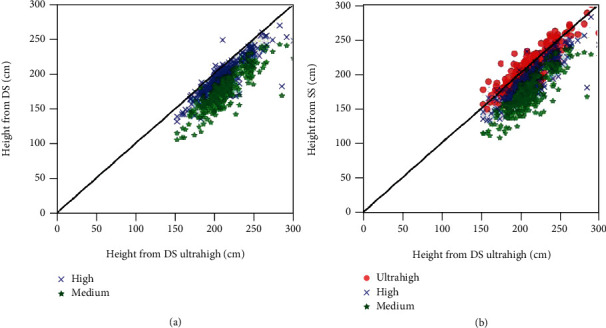
Comparison of plant height from (a) DS and (b) SS flights with the reference plant height from DS ultrahigh dense clouds.

Ideally, the plant height should be validated with reference from perfectly coregistered high-resolution laser scanning data or manual height measurements as mentioned in other studies. However, these independent reference measurements were not available over the breeder's commercial production fields used in this study. The DS with an ultrahigh quality level will be used here as the reference since it provides the best georeferencing accuracy as demonstrated earlier. The plant height computed from DS and SS shows good correspondence with the reference plant height on all quality levels (*R*^2^ > 0.65, Figure 7 and Table 5). The smallest difference was observed on ultrahigh quality point clouds from SS flight (RRMSE = 6.5% and bias = −4.8 cm). When the quality level degraded, larger RRMSE and bias were observed. This was consistent with results found in the 3D dense cloud comparison as shown in Table 4. With the same quality level, the plant height calculated from DS flight presents slightly better performances compared to correspondences from SS flight (RRMSE = 9.5% vs. 12.4% for high quality and 18.1% vs. 19.5% for medium quality).

## 4. Discussion

### 4.1. DS Configuration Improves Both Acquisition and Processing Efficiency

Under the DS configuration, the high correlation between the far green images (*f* = 4.2 mm) and the green images (*f* = 8 mm) and the large across-track and along-track overlaps provided by the shorter focal length allowed to align efficiently all the bands, including the ones with *f* = 8 mm that have much lower across-track and along-track overlap. The alignment process is achieved thanks to the robust photogrammetry algorithms and was found successful over more than one hundred flights taken with the DS configuration.

The improved efficiency of the DS configuration appears appealing as compared to that of the SS configuration, without compromising the spatial resolution. While the processing efficiency is probably not the main advantage of the DS configuration, the acquisition efficiency is particularly relevant to ease the scanning of large experimental fields for which the limited autonomy of the batteries can require several flights, thus increasing the total acquisition time. The radiometric calibration of the data can also benefit from the improved acquisition efficiency because uncertainties due to possibly stronger variations in the sun position or the environmental conditions (cloud shadow, wind) during the flights can be limited.

This technique could be easily extended to other cameras (e.g., RGB cameras) and combinations of other focal lengths. Although this study presents results on a maize field, we successfully applied it over multiple crops, such as wheat, rapeseed, sunflower, and sugar beet, which shows the independence to the crop type.

### 4.2. The DS Configuration Lowers the Georeferencing Error

Our results demonstrate that the DS configuration that combines the 4.2 mm and the 8 mm focal length improves the geometric accuracy, especially in the *z*-dimension, and provides denser 3D point clouds. The addition of oblique views taken by the 4.2 mm camera, by introducing more directional information and redundancy, allows to derive the radial lens distortion more accurately during the bundle adjustment phase of the SFM algorithm and may also compensate for an insufficient and/or not well-distributed set of ground control points. These results are consistent with previous findings based on the comparison between the different acquisition schemes (nadir, oblique, and nadir and oblique) against terrestrial laser scanning data over buildings or complex terrains. These studies showed that coupling nadir and oblique imagery can significantly decrease the georeferencing error, improve the precision, and reduce point cloud data gaps [6, 22, 39].

More recently, [23, 36] proposed to fly both with nadir and oblique cameras to build the 3D dense cloud using the SfM algorithm without ground GCPs. This will offer the advantage to simplify the UAV flight preparation process and thus increase the acquisition efficiency.

### 4.3. Ultrahigh Quality of the 3D Dense Cloud Should Be Used to Estimate Plant Height

An important point addressed in this study was to investigate the impact of the dense cloud quality on plant height estimation.

Plant heights retrieved from DS or SS configurations show differences depending on the quality level of the 3D point clouds. The dense cloud is critical when modeling the 3D structure of crops from the SfM algorithm. Its density and accuracy depend on the sensor, flight altitude, and processing quality level. The key factor to control the dense cloud is the quality selected when building it. Few studies have been conducted to evaluate the impact of the dense cloud quality on plant height estimations using an RGB camera. For example, [40] found marginal differences of 3.3 cm between high and medium quality dense clouds generated from RGB images in forests and finally decided to use the medium quality processing workflow because of the lower computational cost. [21] compared the use of high and ultrahigh quality dense clouds to compute plant height and found that the high quality dense cloud showed surprisingly better performances than the ultrahigh quality one. [6, 20] showed that RGB sensors with a GSD better than 1 cm provide satisfactory crop height estimates with a 3D dense cloud at medium or high quality. Conversely, results from this study indicate that images acquired with multispectral cameras that usually have lower spatial resolutions and larger GSD (GSD > 2 cm) have to be processed at high or ultrahigh quality to get a sufficiently dense point cloud. For example, [15] estimated plant height over barley using ultrahigh quality to compute the 3D dense cloud derived from a hyperspectral imager with a degraded spatial resolution (21 cm GSD at 30 m altitude).

In this study, we only used the visible bands to compute plant height. However, this trait could benefit from the spectral information provided by the multispectral camera, particularly for the separation between soil and vegetation points: the near-infrared bands that offer a better contrast between the vegetation and the background could improve the accuracy with which the DEM is retrieved from the 3D point cloud, with positive consequences on plant height estimates [21]. More investigations are therefore necessary to better use the spectral information for plant height estimations.

## 5. Conclusions

We proposed to use an imaging system where two different focal length lenses were used concurrently aboard a UAV. In our case, a six-band multispectral camera with relatively low resolution (1.3 Mpix) had one band equipped with a 4.2 mm focal length lens providing a wider swath than the five other bands equipped with an 8 mm focal length lens. The wider swath optics increased the coverage capacity, allowing to decrease the flight time by a factor of two (from 24 min to 13 min) or increase by the same factor the area to be sampled within a single flight, while keeping the same spatial resolution for the other five bands with a narrower swath. The increased image acquisition efficiency of the double swath (DS) configuration was also associated with an increase of the image processing efficiency (135 min of DS vs. 455 min of SS for ultrahigh quality dense cloud) because of the reduced number of images (1896 images of DS vs. 2868 images of SS) to process when flying with a wider swath camera. We demonstrated that combining wide and narrow swath optics improved the georeferencing accuracy of the 3D dense cloud by 5.6 cm as compared to the use of narrow swath bands only. The best improvement was found for the *Z* dimension (6 cm) thanks to the availability of larger view angles (up to 35.3°). Application to estimate plant height over a maize field from 52 m altitude showed that better performances were obtained as compared to the standard use of narrower swath optics only. However, we also showed the importance of the quality selected when generating the 3D point cloud. In our case, significantly better performances were observed for the ultrahigh quality level.

## Figures and Tables

**Table 1:** Characteristics of the flights for the SS and DS configurations.

		SS	DS
Multispectral camera	Along-track overlap for *f* = 8 mm	80%	62%
	Across-track overlap for *f* = 8 mm	80%	62%
	Along-track overlap for *f* = 4.2 mm	90.5%	80%
	Across-track overlap for *f* = 4.2 mm	89.5%	80%
	Number of multispectral images	992	487
Flight duration (minutes)		24	13

**Table 2:** Summary of the efficiency assessment of DS and SS flights. Time is expressed in minutes.

	SS	DS	DS/SS (%)
Flight duration (min)	24	13	54
Bundle adjustment			
Number of images	2868	1896	66
Number of tie points	2,433,055	1,826,117	75
Alignment matching time (min)	31	21	68
Alignment time (min)	40	25	63
3D dense cloud			
3D dense cloud processing time (min)			
*Medium quality*	29	8	28
*High quality*	98	26	27
*Ultrahigh quality*	384	89	23
Density of points^∗^ (pts/m^2^)			
*Medium quality*	152	168	111
*High quality*	931	953	102
*Ultrahigh quality*	5393	4893	91
Total processing time (ultrahigh quality) (min)	455	135	30

^∗^This is the number of dense cloud points inside the field boundary divided by the field area.

**Table 3:** Georeferencing errors for the DS and SS configurations over an orthomosaic of 2.4 cm resolution computed with ultrahigh quality level.

Flight		GCP									Average
		1	2	3	4	5	6	7	8	9	
DS	*δ*_*X*,*i*_	*2.0*	*0.5*	*0.2*	*0.1*	*-0.1*	*-0.8*	*0.3*	*-0.7*	*-1.5*	*0.9*
	*δ*_*Y*,*i*_	*0.7*	*0.1*	*-2.1*	*3.6*	*-0.3*	*-2.8*	*1.4*	*0.9*	*-1.0*	*1.8*
	*δ*_*Z*,*i*_	*1.6*	*-0.8*	*-1.8*	*0.8*	*-0.5*	*-1.3*	*1.7*	*0.3*	*-2.2*	*1.4*
	*ε*_*i*_	*2.7*	*0.9*	*2.7*	*3.7*	*0.6*	*3.2*	*2.2*	*1.2*	*2.8*	*2.4* ^∗^
SS	*δ*_*X*,*i*_	*1.2*	*1.3*	*2.3*	*1.5*	*-0.8*	*1.0*	*-0.5*	*-1.3*	*-0.1*	*1.2*
	*δ*_*Y*,*i*_	*-2.2*	*2.1*	**-5.3**	*3.8*	*0.1*	*-2.9*	*2.8*	*-1.0*	*-2.5*	*2.9*
	*δ*_*Z*,*i*_	*1.2*	*1.9*	**10.6**	*-3.7*	**6.2**	**-11.8**	**-6.9**	**7.1**	**-9.1**	**7.4**
	*ε*_*i*_	*2.8*	*3.1*	**12.0**	**5.5**	**6.2**	**12.2**	**7.5**	**7.3**	**9.4**	**8.0** ^∗^

^∗^Overall error *δ*. Italic texts correspond to an acceptable georeferencing error while boldface texts are considered nonacceptable.

**Table 4:** Median and standard deviation of M3C2-calculated distance (cm) between the 3D dense clouds computed either from DS or SS with different qualities (ultrahigh, high, and medium).

	Median	Standard deviation
DS (ultrahigh)-SS (ultrahigh)	-1	19
DS (high)-SS (high)	-5	23
DS (medium)-SS (medium)	-9	25
DS (high)-DS (ultrahigh)	-9	27
DS (medium)-DS (ultrahigh)	-11	27
SS (high)-SS (ultrahigh)	-14	34
SS (medium)-SS (ultrahigh)	-20	27

**Table 5:** *R* ^2^, bias, RMSE, and relative RMSE (RRMSE) between plant heights derived from DS or SS configurations with plant height from DS ultrahigh dense clouds used as the reference.

Flight	Quality level	*R* ^2^	RMSE (cm)	RRMSE (%)	Bias (cm)
DS	Ultrahigh	1.00	0.0	0.0	0.0
	High	0.76	20.2	9.5	-15.9
	Medium	0.71	38.5	18.1	-35.7
SS	Ultrahigh	0.75	13.9	6.5	-4.8
	High	0.72	26.4	12.4	-22.6
	Medium	0.67	41.4	19.5	-38.3

## Data Availability

The data used for this paper is freely available upon permission of HIPHEN.

## Supplementary Materials

Supplementary Materials Table S1: the number of images covering each GCP for double swath and single swath flights on 530 nm and 570 nm wavelengths. The number of images was computed based on orthoimage of each flight in the spatial resolution of 50 cm.
