# Supplementary material for: A Double Swath Configuration for Improving Throughput and Accuracy of Trait Estimate from UAV Images
Source: Plant Phenomics. 2021 Dec 6;2021:9892647. doi: 10.34133/2021/9892647 (PMC8672205; doi:10.34133/2021/9892647)
Supplement: Supplementary Materials — Table S1: the number of images covering each GCP for double swath and single swath flights on 530 nm and 570 nm wavelengths. The number of images was computed based on orthoimage of each flight in the spatial resolution of 50 cm. [file 9892647.f1.docx]

Supplementary Materials

|  | GCP | 1 | 2 | 3 | 4 | 5 | 6 | 7 | 8 | 9 |
| --- | --- | --- | --- | --- | --- | --- | --- | --- | --- | --- |
| Double swath | 530 nm | 16 | 18 | 12 | 12 | 10 | 8 | 34 | 19 | 15 |
|  | 570 nm | 62 | 58 | 35 | 56 | 61 | 34 | 70 | 60 | 39 |
|  | Sum | 78 | 76 | 47 | 68 | 71 | 42 | 104 | 79 | 54 |
|  | | | | | | | | | | |
| Single swath | 530 nm | 66 | 75 | 93 | 75 | 81 | 69 | 90 | 84 | 78 |

***Table S1. The number of images covering each GCP for double swath and single swath flights on 530 nm and 570 nm wavelengths.*** *The number of images were computed based on orthoimage of each flight in the spatial resolution of 50 cm.*
